# Supplementary material for: Low Hysteresis and Fatigue-Resistant Polyvinyl Alcohol/Activated Charcoal Hydrogel Strain Sensor for Long-Term Stable Plant Growth Monitoring
Source: Polymers (Basel). 2022 Dec 26;15(1):90. doi: 10.3390/polym15010090 (PMC9824891; doi:10.3390/polym15010090)
Supplement: Supplementary file 1 [file polymers-15-00090-s001.zip › polymers-2101365-supplementary.pdf]

SUPPLEMENTARY INFORMATION FOR

Low Hysteresis and Fatigue-Resistant Polyvinyl Alcohol/Activated Charcoal Hydrogel Strain Sensor for Long-Term Stable Plant Growth Monitoring

Lina Wang [1,2,†], Zhilin Zhang [1,2,†], Jie Cao [1,†], Wenqian Zheng [1,2], Qi Zhao [1], Wenna Chen [2], Xinye Xu [1,2], Xiaoyu Luo [1,2], Qi Liu [1,2], Ximei Liu [1,2], Jingkun Xu [1,3,*], Baoyang Lu [1,2,*]

[1] Jiangxi Key Lab of Flexible Electronics, Flexible Electronics Innovation Institute, Jiangxi Science and Technology Normal University, Nanchang 330013, China

[2] School of Pharmacy, Jiangxi Science and Technology Normal University, Nanchang 330013, China

[3] School of Chemistry and Molecular Engineering, Qingdao University of Science and Technology, Qingdao 266042, China

* Correspondence: xujingkun1971@yeah.net (J.X.); luby@jxstnu.edu.cn (B.L.); Tel.: +86-791-88537967 (B.L.)

† These authors contributed equally to this work.

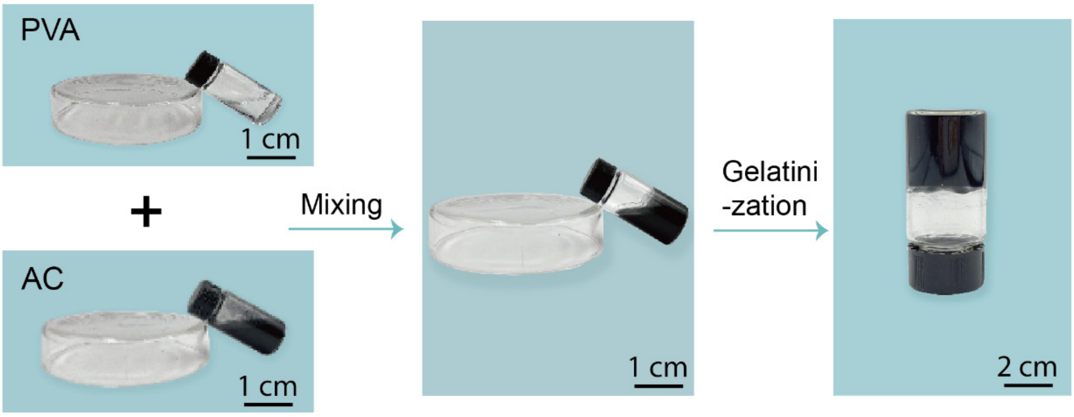

Figure S1: Photograph of the PVA-AC hydrogel preparation process.

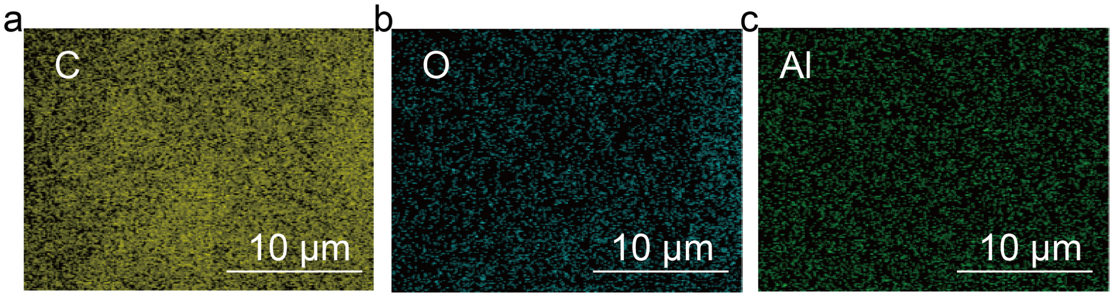

Figure S2: Elemental mapping analysis of PVA-AC from cross-sectional morphologies of Scanning Electron Microscope.

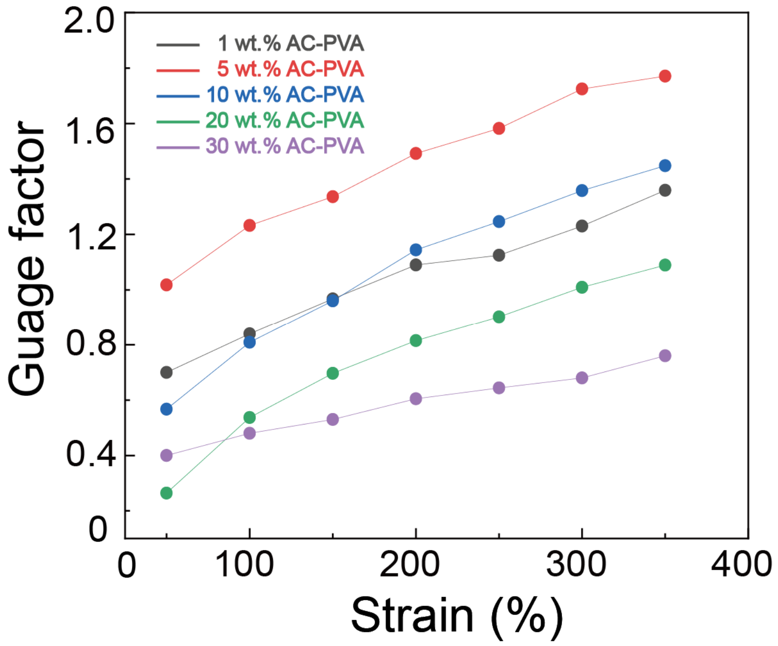

Figure S3: Gauge factor (*GF*) of PVA-AC hydrogel strain sensors with different AC concentrations.

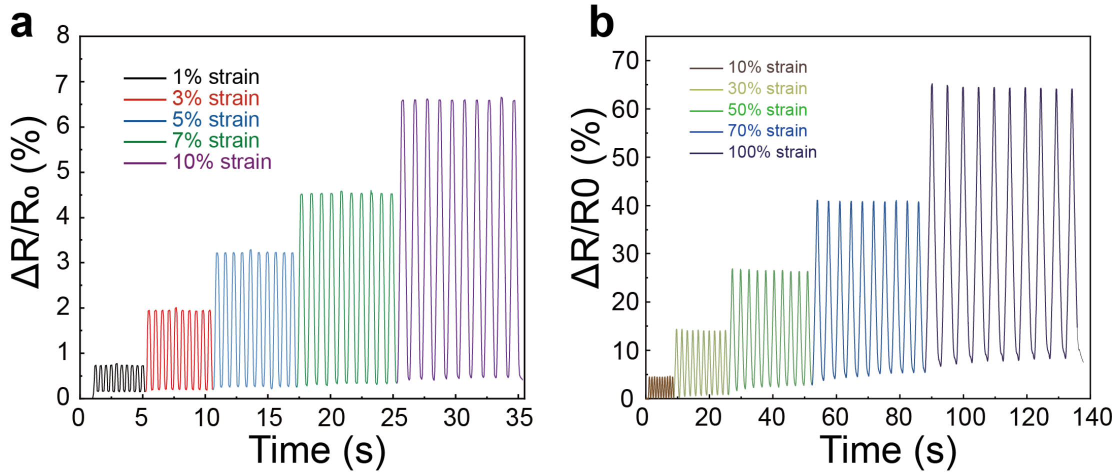

Figure S4. The relative resistant change of the PVA-AC conducting hydrogel strain sensor at the strain of 1%-10% and 10%-100%.
